# Management of urinary tract infections in the community: a clinical audit and patient survey

**DOI:** 10.3399/BJGPO.2022.0191

**Published:** 2023-10-04

**Authors:** Stacey Jayne Fisher, Clive Graham, James Kennard, Leon Jonker

**Affiliations:** 1 Research & Development Department, North Cumbria Integrated Care NHS Foundation Trust, Penrith, UK; 2 Microbiology Department, North Cumbria Integrated Care NHS Foundation Trust, Carlisle, UK; 3 Banbury Cross Health Centre, Banbury, UK; 4 Research & Development Department, North Cumbria Integrated Care NHS Foundation Trust, Penrith, UK

**Keywords:** antibacterial agents, antibiotics, clinical guideline, health confidence, self-management, urinary tract infections, UTI

## Abstract

**Background:**

Urinary tract infection (UTI) is a common ailment but can develop into sepsis. The outcomes related to UTI may potentially be affected by both patient and clinician management of UTI.

**Aim:**

To explore the circumstances around a single UTI episode to determine whether there are patient and clinician-related variables that may contribute to differences in management.

**Design & setting:**

Survey and clinical audit in 12 general practices in England.

**Method:**

Patients (*n* = 504) completed a bespoke survey and their corresponding index UTI consultation was audited. The TARGET (Treat Antibiotics Responsibly, Guidance, Education and Tools) UTI audit toolkit was utilised.

**Results:**

A significantly higher proportion of females compared with males used self-management measures. Increase in fluid intake was 78% for females aged <65 years and 71% for females aged >65 years compared with 53% for males (*P*<0.001, Χ^2^ test). Analgesic use was 50% for females aged <65 years and 41% for females aged >65 years compared with 36% for males (*P* = 0.036, Χ^2^ test). Males also indicated they lacked UTI knowledge when compared with females (*P* = 0.002, Kruskal-Wallis test). Males also claimed to have waited significantly longer for a consultation appointment (*P* = 0.027, Χ^2^ test). Antibiotics were prescribed in 98% of all cases, with adherence to clinical diagnostic guidelines lowest in females aged <65 years. Only 40% (89/221 of cases in this guideline sub-cohort [females aged >65 years]) would have been a UTI, according to TARGET criteria, following a medical record audit.

**Conclusion:**

UTI symptom management by clinicians is suboptimal; the presence or absence of symptoms is often insufficiently recorded in medical records. Additionally, suboptimal adherence to guidelines concerning urinalysis and microbiological investigation is common. Known increased clinical risks for males may be compounded by their more limited knowledge of (self)-managing UTI and their comparatively late presentation.

## How this fits in

Urinary tract infection (UTI) is managed differently depending on patient sex and age, due to differences in associated risk of complications. Male patients indicate being less knowledgeable of UTIs, utilise self-management remedies less often, and present later to a healthcare professional. For this cohort of patients, sub-optimal clinical management of UTIs was identified; this may compromise patient safety and antimicrobial stewardship. Public health interventions aimed at males are indicated to ultimately reduce the risk of UTI complications and sepsis.

## Introduction

Of all bacterial infections managed in primary care in developed countries, UTI is one of the most common.^1,2^ Serious complications owing to sepsis can occur; therefore, to mitigate that risk, the rate of antibiotic prescription tends to be high.^3–5^

The initial management of UTI, by both patient and healthcare professional, may influence clinical outcomes. Through interviews with a small cohort of patients, Lecky and colleagues^6^ identified a need for enhanced patient–clinician shared decision making with a focus on self-care, safety netting, and preventive advice. If an accurate overview of the circumstances around initial UTI diagnosis and treatment can be established, key areas of focus may be determined to optimise (self-) care. Clinical guidance for UTI has been developed by the Royal College of General Practitioners (RCGP), including the TARGET initiative to aid GPs with management of UTIs in the community.^7^ TARGET can be utilised to check for adherence to best practice.^8^ The aim of this project was to evaluate the circumstances around a patient’s own behaviour and initial management of their UTI symptoms. This evaluation was then matched with the resulting index consultation with a healthcare professional (audited using the TARGET tool) to determine whether there were any pre-consultation behaviours (for example, self-help measures) that demonstrated a significant relationship with their presenting symptoms, management plan, and illness outcomes. Together, this may highlight areas for improvement of care for patients and healthcare professionals alike.

## Method

### Study design and patients

This study involved a combination of a patient postal survey and a subsequent clinical audit of the index UTI episode for those patients returning a completed survey (see Supplementary Figure S1). The study was conducted between September 2021 and October 2022 in 12 different general practices in England. Invited patients were those aged 18–80 years with diagnosis of (suspected) community-acquired UTI or use of nitrofurantoin, trimethoprim, or pivmecillinam antibiotics for UTI recorded in their medical records within the past 6 months. The exclusion criteria included lack of mental capacity, or other significant medical (for example, acute hospitalisation, or palliative care needs) or social issues (for example, care home resident), and the use of a urinary catheter. Informed consent comprised of the patient returning the completed survey and acknowledging their medical records would be audited for index UTI episode.

### Survey and audit outcome measures

The patient survey included questions regarding self-management before presenting to a healthcare professional, symptoms associated with the UTI, and interaction with the healthcare professional. The Health Confidence Score was included to measure patients’ self-reported UTI knowledge; it has been applied previously in a genitourinary patient population.^9,10^ The clinical audit of the index UTI episode for participating patients was performed with TARGET UTI.^7^ National Institute for Health and Care Excellence (NICE) guidelines advising antibiotic choice for UTI were also consulted.^5^ The relevant audit tool and guideline was used for men and women aged <65 years and aged >65 years. All audits were undertaken by two GPs.

### Statistical analyses

A minimum overall survey sample of 167 responses was required to achieve a confidence level of 99% and a margin of error of 10%. Data were initially processed using Excel (Microsoft) and analysed with Statistics Package for the Social Sciences (SPSS; version 24). Inferential analyses were applied as indicated in the Results section (*P*<0.05 was deemed statistically significant). For binary and nominal data, Χ^2^ tests were applied. For ordinal data from the Health Confidence Score categories, Kruskal–Wallis test was applied. Binary logistic regression was used to evaluate if any variables were associated with the binary result of the outcome variable, with Nagelkerke *R* ^2^ (maximum achievable value is 1, that is, 100%) used to determine the variance contributed by the variables to the outcome variable. All inferential statistical tests were intended to explore and quantify any differences and associations between variables, rather than aimed at testing predefined hypotheses. Surveys with more than two missing answers were excluded; for a missing answer the mode (binary data) or median (ordinal data) answer was determined and imputed.

## Results

An initial 2655 patients were initially identified, 1792 (68%) were deemed eligible and sent a postal survey. Of the invitees, 525 patients returned their survey (response rate 29%). Survey responders had a mean age of 61 years (84% female), whereas the mean age for invited patients was 53 years (85% female). The reasons for excluding 21 surveys were patients having left the general practice (*n* = 16) and incomplete returned surveys (*n* = 5). For 50 out of 504 analysed surveys, the missing answer to <2 questions had to be added. Sex distribution was as follows: 81 males (30: aged <65 years and 51: aged >65 years) and 423 females (221 aged: <65 years and 202 aged: >65 years). A total of 482 (96%) consulted a general practice within office hours whereas 17 (3%) consultations were during GP out-of-hours cover, and 5 (1%) attended accident and emergency (A&E). From the 504 consultations, 157 (31%) were face to face, 318 (63%) via telephone, and 9 (6%) via email or text*.* Doctors managed 336 (67%) patients whereas nurses and allied health professionals managed 168 (33%) patients. A significant difference in distribution among sub-cohorts was observed for mode of consultation; females aged <65 years were consulted relatively more via text and males were seen more frequently face to face (*P*<0.001, Χ^2^).

Table 1 outlines the self-management measures reported by patients. A significantly higher proportion of females, particularly those aged <65 years, used self-management measures. Increase in fluid intake was 78% for females aged <65 years and 71% for females aged >65 years compared with 53% for males (*P*<0.001, Χ^2^ test). Analgesia use was 50% for females aged <65 years and 41% for females aged >65 years compared with 36% for males (*P* = 0.036, Χ^2^ test).

**Table 1.** Patient self-management and hygiene control

Item	Male (*n* = 81), yes (% total)	Female <65 years (*n* = 221), yes (% total)	Female >65 years (*n* = 202), yes (% total)	*P*-value (Χ^2^)
Increase in fluid intake	43 (53)	172 (78)	144 (71)	<0.001
Use of over-the-counter cystitis remedies	6 (7)	89 (40)	56 (28)	<0.001
Use of analgesia	29 (36)	111 (50)	82 (41)	0.036
Use of cranberry products	16 (20)	95 (43)	68 (34)	0.001
Sought advice from a pharmacist	5 (6)	28 (13)	17 (8)	0.160

**Figure 1.**
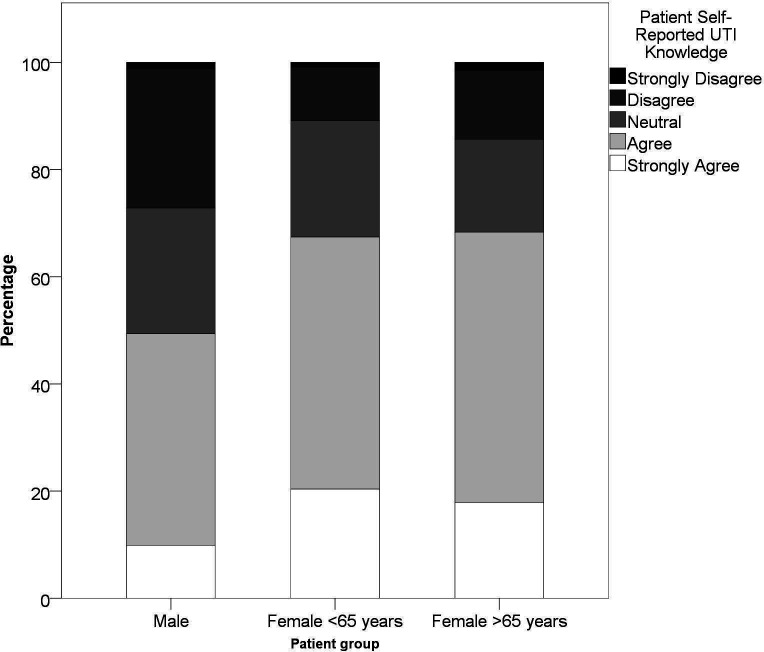
Patient feedback on their agreement with the statement: ‘*I know enough about urinary tract infections.'* Male patients agreed significantly less with the patient confidence question on the theme of ‘knowledge’ than female patients of any age (*P* = 0.002, Kruskal–Wallis test; total *n* = 504)

Patients’ confidence in relation to managing their UTI was explored with the Health Confidence Score that included questions on knowledge (*‘I know enough about UTI’*), self-management (‘*I can look after a UTI when I get one*’), help-seeking (‘*I can get the right help for treatment of a UTI if I need it*’), and decision involvement (‘*I am involved in decisions about managing and treating a UTI*’). There was a significant difference in opinion between males, females aged <65 years, and females aged >65 years when it concerned ‘knowledge’ (*P =* 0.002, Kruskal–Wallis test; see Figure 1). Whereas there was no significant difference when comparing the following remaining three themes: ‘self-management’ (*P =* 0.063), ‘help-seeking’ (*P =* 0.70), and ‘decision involvement’ (*P =* 0.40).

**Figure 2.**
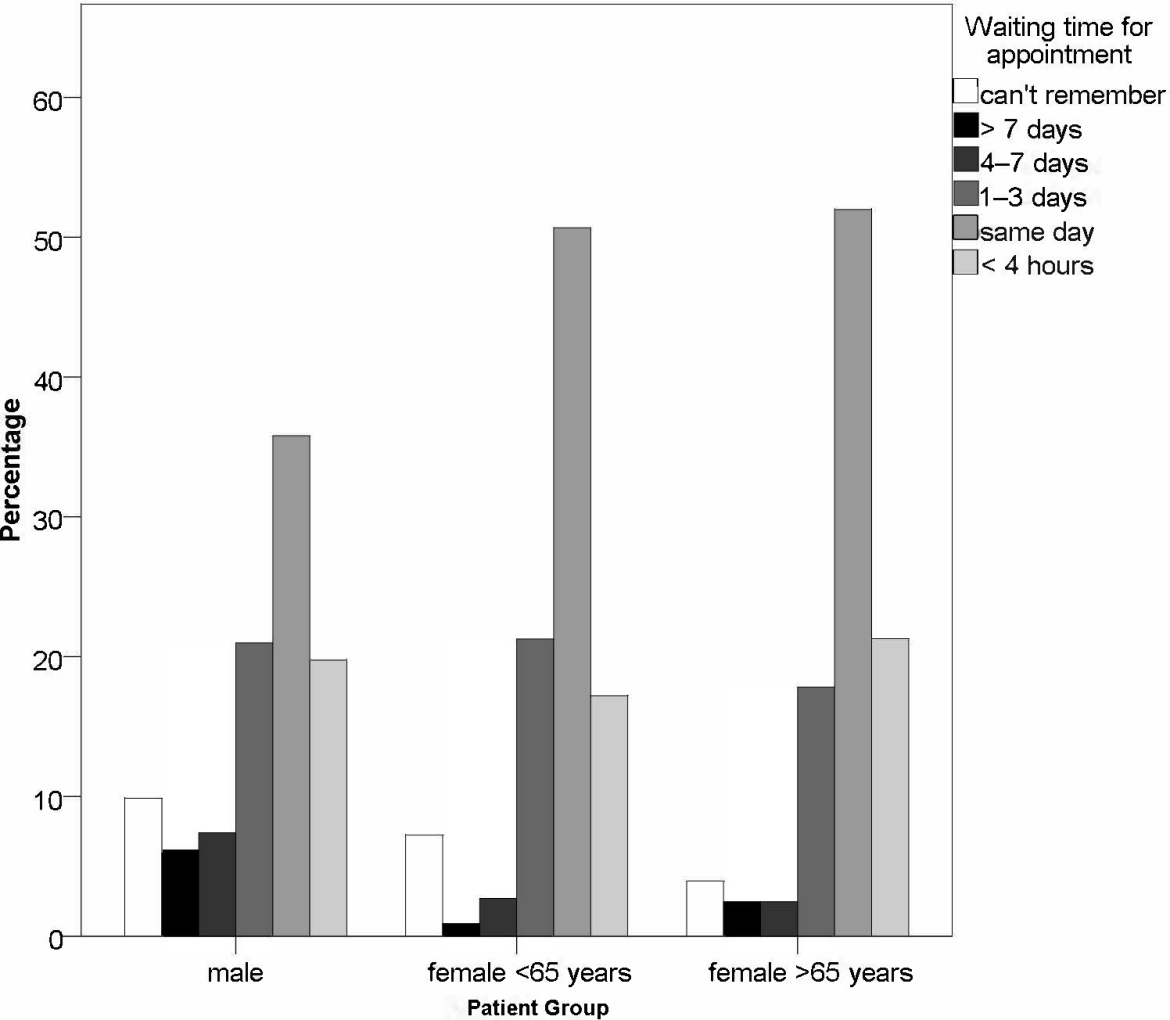
Patient feedback on how long they had to wait for an appointment for their UTI. Male patients indicated that they waited longer for an appointment than female patients of any age (*P* = 0.027, Χ^2^ test; total *n* = 504)

Among the three sub-cohorts, there was no notable difference in number of days the symptoms were present before a patient decided to contact a healthcare professional. On a Likert scale of ‘same day’/‘1–3 days’/‘4–7 days’/‘more than 7 days’, the median time for each sub-cohort was 4–7 days (*P* = 0.60, Χ^2^ test). However, there was a significant difference in patient-reported waiting time for having the actual consultation (*P* = 0.027, Χ^2^ test), see Figure 2. Male patients claimed to have waited longer for an appointment.

The recording of presence or absence of UTI symptoms is an essential element of clinical diagnostics. There was a low degree of agreement between patients and clinical staff for four different hallmark UTI symptoms, see descriptive summary in Table 2. It should be noted that the clinician’s entry in the medical notes was prospective, whereas the patient’s was a retrospective recall of a consultation up to 6 months’ ago. According to the source medical records, patients were issued general information on how to manage their symptoms in 24% (*n* = 121/504) of cases, patients were safety-netted 61% of the time (*n* = 308/504), and were issued the TARGET UTI patient information leaflet or weblink 2% of the time (*n* = 10/504).

**Table 2.** Comparison of patient recall and clinician-recorded incidence of different symptoms associated with UTI

Symptom	Patient recorded as symptom present?	Recorded in medical records (*N* = 504), *n* (%)		
		Symptom not recorded (as either present or absent)	Symptom confirmed absent	Symptom confirmed present
Dysuria	No	47 (9)	18 (4)	62 (12)
	Yes	106 (21)	7 (1)	264 (52)
				
New nocturia	No	223 (44)	3 (0.6)	26 (5)
	Yes	200 (40)	1 (0.2)	51 (10)^a^
				
Cloudy urine	No	257 (51)	7 (1)	24 (5)
	Yes	153 (30)	4 (1)	57 (11)^a^
				
Frequency	No	53 (11)	3 (0.6)	69 (14)
	Yes	147 (29)	2 (0.4)	230 (46)^a^

^a^Instances where both patient and clinician noted presence of the symptom

Table 3 shows the audit of the patient medical records using TARGET and NICE guidelines; 59% of males (*n* = 48/81), 40% of females aged <65 years (*n* = 89/221) and 69% of females aged >65 years (*n* = 135/202) were correctly diagnosed as having a UTI. Pyelonephritis was adequately assessed in only 22% (*n* = 112) of cases and sepsis in 32% (*n* = 161) of cases. In females aged <65 years, urine dipsticks (urinalysis test strips) were often not conducted when indicated, for instance when the patient had <1 urinary symptom in order to appropriately diagnose a UTI (See Table 3). Conversely, urine dipstick was often performed in females aged >65 years when not indicated. Mid-stream urine (MSU) microbiological culture was frequently not submitted for analysis for both males and females aged >65 years when this was indicated (68% for both groups). Antibiotics were prescribed for 98% of all patients who consulted a healthcare professional for a possible UTI. When an antibiotic was prescribed (putting aside whether or not it was indicated according to the TARGET audit toolkit), which occurred in 495 out of 504 cases (98%), the choice of antibiotic was correct in 89% of cases (*n* = 439/495), the dosage in 98% (*n* = 484/495) and the course length in 85% of cases (*n* = 422/495), respectively, and in line with NICE antibiotic guidelines for UTI. The number of cases with correct antibiotic course length included 29 justified deviations (7% of total number prescriptions).

**Table 3.** Clinical audit against TARGET UTI audit toolkit and NICE antibiotic (for UTI) guidelines

Item	Male (*N* = 81), yes, *n* (%)	Female <65 years (*N* = 221), yes**, *n* (%**)	Female >65 years (*N* = 202), yes**, *n* (%**)	*P*-value (Χ^2^)
Genitourinary causes adequately assessed^a^	6 (7)	24 (11)	16 (8)	0.490
Pyelonephritis adequately assessed^a^	23 (28)	46 (21)	43 (21)	<0.001
Sepsis adequately assessed^b^	33 (41)	68 (31)	60 (30)	<0.001
Correct UTI diagnosis otherwise made^a^	48 (59)	89 (40)	135 (69)	<0.001
Correct urine dipstick analysis^a^	45 (56)	102 (46)	146 (72)	<0.001
Correct MSU microbiological culture management^a^	55 (68)	150 (68)	107 (53)	0.003
Antibiotics prescribed^b^	80 (99), of which *n* = 2 delayed	218 (99), of which *n* = 3 delayed	197 (98%), of which *n* = 8delayed	0.430

^a^In accordance with TARGET UTI audit guidance. ^b^In accordance with NICE UTI antibiotic guidelines.

MSU = mid-stream urine. NICE = National Institute for Health and Care Excellence. TARGET = Treat Antibiotics Responsibly, Guidance, Education and Tools. UTI = urinary tract infection.

Binary logistic regression analyses were conducted to further explore if certain outcome variables were associated with consultation-related variables. Table 4 shows the variables associated with the diagnosis of UTI based on adherence to clinical guidelines. The results indicate that clinical guidelines were followed less often by GPs, in case of patients aged <65 years, and when the consultation was conducted by telephone. UTI was appropriately diagnosed in 50% (*n* = 168/336) of GP consultations and 65% (*n* = 109/168) of nurse and allied health professional (AHP) consultations, respectively. Similarly, a written assessment for sepsis and pyelonephritis was conducted significantly less often by GPs compared with nurses and AHPs (*P*<0.001, Χ test), and more often outside standard general practice hours and settings. Furthermore, these assessments were most often made when the consultation was face to face (see Supplementary Table S1). Sepsis was assessed in 81 of 336 (24%) cases by GPs and in 80 of 168 (48%) cases by nurses and AHPs. The regression models for both sepsis and pyelonephritis as outcome variable showed weak associations, with the type of staff, consultation and patient accounting for 19% and 15% of the variance in sepsis or pyelonephritis, respectively. Finally, Supplementary Table S2 shows that any associations between the above mentioned variables and the outcome variables ‘correct urinalysis’ and ‘correct MSU microbiological culture’ are very weak, based on the Nagelkerke *R* ^2^ values for the models (accounting for 11% and 6% of variance, respectively). Only the type of patient (as split by clinical guideline) is associated with difference in good practice on this front, as also shown in Table 3.

**Table 4.** Binary logistic regression analysis to determine if variables are associated with correct assessment for UTI by clinical staff

Variable	*P*-value	Odds ratio	95% CI	Interpretation
Staff role (GP versus nurse or AHP)	<0.001	0.46	0.30 to 0.71	GP consultation less often associated with correct UTI assessment
Setting (GP OOH or hospital versus GP in-hours)	0.130	2.15	0.79 to 5.85	NSA
Male patients	<0.001			Correct assessment of UTI more common in males and females aged ≥65 years compared with females aged <65 years
Female patients aged <65 years	0.550	0.84	0.47 to 1.49	
Female patients aged ≥65 years	<0.001	0.37	0.24 to 0.58	
Consultation mode: text	0.018			Text message consultations more often associated with correct UTI assessment
Consultation mode: telephone	0.037	2.70	1.06 to 6.89	
Consultation mode: face to face	0.270	0.78	0.50 to 1.21	
Correct urine dipstick application	<0.001	0.27	0.18 to 0.41	Correct application of urine dipstick significantly linked to correct UTI assessment
Correct MSU microbiological culture management	0.150	1.35	0.90 to 2.03	NSA
Nagelkerke *R* ^2^ value for model	0.240			

AHP = allied health professional. MSU = mid-stream urine. OOH = out of hours. NSA = no significant association. OOH = out of hours. UTI = urinary tract infection

## Discussion

### Summary

The study has suggested that the degree of knowledge and familiarity that a patient has of UTIs may influence how they self-manage and consult healthcare professionals. The study has identified significant evidence suggesting male patients are less likely to try to manage symptoms themselves and they delay consulting a healthcare professional. Furthermore, across all patients, a lack of documentation of absence or presence of symptoms and an inappropriate use of both dipstick urinalysis and MSU microbiological culture by clinical staff appear to coincide, which may negatively impact on clinical guideline adherence.

### Strengths and limitations

A large sample of patients were invited to participate in this study and therefore the cohort was able to be stratified by sex and age-specific UTI clinical guideline. The sample size for the male cohort was smaller, although UTIs are less common in males than in females. Males were included in this study and the focus was on patients who were living independently and not living in a residential or care home, in contrast to previous studies.^4,6,11^ The response rate of just under 30% for the surveys is lower than reported for studies conducted face to face in the general practice but near-identical to a recent postal survey study conducted in the same general practices.^12,13^ Non-responder bias may therefore be a risk and limit generalisability of the findings;^14^ the average age of survey responders, for instance, was higher than for those invited to complete the survey. The intention and strength of the study was to be able to cross-reference patient feedback and medical records for an index UTI episode. Validated measures were used where possible for the patient survey and the audit.^7,9^ Reliance on GP documentation and patient’s memory of their consultation (up to 6 months previously) may not give the complete picture of what happened in real-time during the consultation. It is plausible GPs did adequately assess UTI symptoms but failed to document this, despite negative findings being as important as positive ones. Outcome measures used in previous papers on the topic were deployed too, such as self-treatment options used by patients, as described by Butler and colleagues.^4^

### Comparison with existing literature

There is little available literature investigating the patient’s circumstances before consulting about their UTI symptoms. Of patients presenting with UTI symptoms, Butler *et al* recorded the use of cranberry juice, the number of days of symptoms, and number of days off work, but the corresponding discussion of these was limited.^15^ The patient’s view of having and managing a UTI has been explored in isolation in a number of studies. One — involving just females in the UK — found that virtually all women (95%) sought advice from a healthcare professional.^4^ In that cohort, the majority of patients consulted a general practice yet a substantial 13% contacted a pharmacist; the latter was also observed in the present study's data (see Table 1). The present study's sub-cohort of female patients took similar self-management steps, as reported previously for the female cohort in the Butler *et al* study. An assessment of the validity of these measures — which in the case of, for example, hydration and cranberry product consumption for the treatment rather than prevention of UTIs is debatable^16,17^ — was beyond the scope of this study. The TARGET patient information leaflets do recommend hydration to all patients and do highlight the lack of evidence for cranberry products.^18^ The provision of the TARGET UTI patient information by clinical staff was very rare in this cohort and does not seem to be an established practice, as observed in another study.^19^ The rates for clinical staff providing generic symptom management and also safety-netting advice were near identical to those observed by others.^4,19^

Poor recording of the absence or presence of UTI symptoms, as well as often inappropriate use of dipstick urinalysis and mid-stream urine (MSU) microbiological culture, contributed to low level of compliance with antibiotic stewardship in this cohort. In another study that audited cases using the TARGET tool, higher compliance was found.^19^ Although the present study started when there were still some severe acute respiratory syndrome coronavirus 2 (SARS-CoV2) restrictions in place, meaning some consultations were not face to face when usually they would have been, it is recognised that variability in clinical practice is a long-standing issue.^20^ Furthermore, the ability to record symptoms should not be affected by the mode of patient consultation. The finding that males are less knowledgeable about the UTI condition and are less pro-active to self-manage is concerning since (older) males are more prone to septicaemia.^21^ It cannot be concluded if the finding of more male patients being seen face to face in this study sample is an active mitigation practised by general practice staff or if it is a result of male patients preferring to be seen in this manner (and it therefore inadvertently contributed to delays in being seen by a healthcare professional owing to longer waiting times for face-to-face consultations).

### Implications for practice

Both patient and clinician behaviours regarding general self-help measures and knowledge of points of care access, along with the distribution of UTI-health education materials would benefit from improvement. Different initiatives to improve self-management by males have already shown to have a degree of effectiveness, and may therefore have scope as a wider public health initiative.^22^ Female patients in particular apply self-management measures, such as cranberry product and over-the-counter cystitis product use, despite limited evidence that these may alleviate or treat acute UTI symptoms.^16,17^ During consultation, clinicians can improve information provision to patients such as distributing the TARGET UTI information leaflets. Adjunct professions, such as pharmacists, could contribute in a similar fashion, although the study identified that unfortunately males presently visit such locations less frequently than females.

The different approach required to diagnose and manage UTI depending on patient age and sex appears to be a challenge for healthcare professionals in primary care, despite age and sex-specific national clinical guidelines having been in place for a number of years. Antimicrobial stewardship will be suboptimal if younger females continue to be prescribed antibiotics when they are not indicated; a delay in prescribing may be prudent, as demonstrated in a past randomised controlled trial.^23^ Conversely, although there is increasing evidence to prescribe immediate antibiotics for male patients, insufficient MSU microbiological culture sampling may potentially increase the inappropriate prescription of antibiotics.^21,24^ How feasible it is to achieve improvements in clinical practice in general practices will be the challenge, since it is known that time pressure erodes adherence to clinical guidelines.^6,25^

## Data

For queries regarding data please contact the corresponding author.
